# Potential Quality Evaluation Method for Radix Astragali Based on Sweetness Indicators

**DOI:** 10.3390/molecules20023129

**Published:** 2015-02-13

**Authors:** Ke Li, Fanrong Gao, Zhenyu Li, Xuemei Qin, Haifeng Sun, Jie Xing, Lizeng Zhang, Guanhua Du

**Affiliations:** 1 Modern Research Center for Traditional Chinese Medicine, Shanxi University, No.92, Wucheng Road, Taiyuan 030006, China; E-Mails: like@sxu.edu.cn (K.L.); gaofanrong@163.com (F.G.); lizhenyu@sxu.edu.cn (Z.L.); xingjie@sxu.edu.cn (J.X.); zhangli@sxu.edu.cn (L.Z.); dugh@imm.ac.cn (G.D.); 2 College of Chemistry and Chemical Engineering, Shanxi University, No.92, Wucheng Road, Taiyuan 030006, China; E-Mail: haifeng@sxu.edu.cn; 3 Institute of Materia Medica, Chinese Academy of Medical Sciences, Beijing 100050, China

**Keywords:** Radix Astragali, sweetness indicator, comprehensive evaluation, quality control

## Abstract

Sweetness is a traditional sensory indicator used to evaluate the quality of the popular Chinese herb Radix Astragali (RA). RA roots with strong sweetness are considered to be of good quality. However, neither a thorough analysis of the component(s) contributing to RA sweetness, nor a scientific investigation of the reliability of this indicator has been conducted to date. In this study, seven kinds of sweetness components were identified in RA and a quality evaluation method based on these components was established and used to characterize the quality of 48 RA samples. The sweetness evaluation method of RA was first built based on the sweetness components, and a comprehensive evaluation index commonly used in quality control of RA was also derived, which was based on the contents of four indicators (astragaloside IV, calycosin glucoside, polysaccharides and extracts). After evaluating the correlation of these indexes the results showed that the level of sweetness exhibited a strong positive correlation with the proposed comprehensive index. Our results indicate that sweetness is one of the most important quality attributes of RA and thus provide a scientific basis for the utility of the sweetness indicator in quality assessment of this Chinese herb.

## 1. Introduction

Radix Astragali (RA), the dried root of *Astragalus membranaceus* (Fisch.) Bunge var. mongholicus (Bunge) Hsiao (MG) or A. *membranaceus* (Fisch.) Bunge (MJ), is an important traditional Chinese herb belonging to the family *Leguminosae*. RA has been used as a restorative tonic and dietary supplement for over 2000 years and it is one of the important staple exports of China [1,2,3,4]. RA roots with strong sweetness are considered to be of good quality in Chinese medicine [5,6]. Studies have shown that sweetness of plants comes both from saccharide ingredients and non-saccharide constituents (organic acids, terpenes, steroids, glycoprotein and other nitrogen-containing compounds), such as glycyrrhizin, stevioside, and monellin [7]. Recent research has shown that the sweetness of RA may also come from sugars and betaines [8]. However, neither a thorough analysis of the component(s) contributing to its sweetness, nor a scientific investigation of the traditional sensory indicators has been conducted to date. In this study, the varieties and contents of components contributing to sweetness in RA were determined and a method for evaluation of RA sweetness based on its sweetness component contents was established to characterize the relative sweetness and quality of 48 samples from different plant sources, growth years and regions.

Previous studies showed that flavonoids, saponins, polysaccharides, amino acids and various trace elements are the main active compounds in RA and have used them as indicators for quality evaluation of RA [9,10,11,12,13]. As a result of this, the quality evaluation of RA has so far been multivariate and multi-index [14,15], therefore, a comprehensive evaluation approach based on principal component analysis with the four common quality control indicators listed in the China Pharmacopeia (2010 Edition) (astragaloside IV, calycosin-glucoside, polysaccharides and extracts) was also established to evaluate the quality of RA samples [16]. This approach of integrating the information of multiple indexes and using a comprehensive approach to evaluate the quality of RA is an effective way to solve the problems of differences in the weights of index components and interactions among the components [14,15,16].

Therefore, the paper aims to explore the correlation of sweetness levels and the comprehensive index though calculation of the “sweetness” and comprehensive index of RAs and comparison of the evaluation results using a statistical method, which will provide more scientific evidence for the traditional quality evaluation approach of RA based on sweetness indicators.

## 2. Results and Discussion

### 2.1. Analysis of Sweetness Components of RA

#### 2.1.1. Qualitative and Quantitative Analysis of the Main Chemical Components of “RA Sweetness”

**Figure 1:**
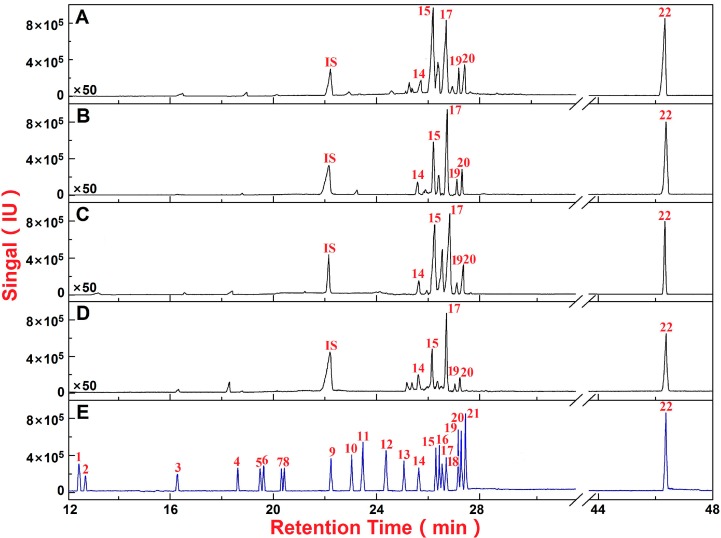
Chromatograms of the total ion current of the acetylated monosaccharide, disaccharide standards and determination of RA samples. Saccharide mappings of the perennial *membranaceus* var. Mongholicus (Bge.) (**A**); fast-growing *membranaceus* var. Mongholicus (Bge.) (**B**); perennial *membranaceus* (Fisch.) Bge. (**C**); fast-growing *membranaceus* (Fisch.) Bge. (**D**); and chromatogram of the total ion current of the acetylated monosaccharide and disaccharide standards (**E**). Peak identities: (1) *meso*-erythritol (RT: 12.43 min, *m/z*: 145, 115, 103); (2) 2-deoxy-d-ribose (RT: 12.76 min, *m/z*: 81, 98, 141); (3) 2-deoxy-d-ribitol (RT: 16.20 min, *m/z*: 159, 103, 117); (4) d-xylopyranose (RT: 18.64 min, *m/z*: 128, 170, 157); (5) L-rhamnopyranose (RT: 19.32 min, *m/z*: 115, 157, 142); (6) L-fucopyranose (RT: 19.60 min, *m/z*: 115, 157, 142); (7) L-arabinopyranose (RT: 20.02 min, *m/z*: 128, 170, 115); (8) l-rhamnitol (RT: 20.25 min, *m/z*: 128, 170, 115); (9) d-ribitol (internal standard, RT: 22.35 min, *m/z*: 115, 145, 103); (10) l-fucitol (RT: 22.81 min, *m/z*: 128, 170, 115); (11) l-arabinitol (RT: 23.02 min, *m/z*: 115, 145, 103); (12) d-xylitol (RT: 23.48 min, *m/z*: 115, 145, 103); (13) d-galactopyranose (RT: 24.32 min, *m/z*: 115, 157, 98); (14) d-glucopyranose (RT: 25.04 min, *m/z*: 115, 157, 98); (15) d-fructopyranose (RT: 25.95 min, *m/z*: 187, 128, 101); (16) d-mannopyranose (RT: 26.17 min, *m/z*: 115, 101, 98); (17) *myo*-inositol (RT: 26.35 min, *m/z*: 168, 115, 126); (18) d-mannitol (RT: 26.58 min, *m/z*: 115, 145, 139); (19) d-sorbitol (RT: 26.91 min, *m/z*: 115, 145, 128); (20) d-dulcitol (RT: 27.09 min, *m/z*: 115, 187, 127); (21) *N*-acetyl-d-glucosamine (RT: 27.26 min, *m/z*: 114, 156, 241) and (22) sucrose (RT: 42.54 min, *m/z*: 169, 109, 211); The IS peak indicates the internal standard.

As shown in Figure 1, Table 1 and Supplementary Table S1, the sweetness of RA can be mainly attributed to the presence of sucrose, glucose, fructose, inositol, sorbitol, dulcitol and betaine. The contents of sucrose and betaine were relatively high, indicating their significant contribution to the taste of RA, while the contents of glucose, fructose, inositol, sorbitol, and dulcitol were low, indicating their minor contribution to the sweetness of RA.

#### 2.1.2. Differences in Sweetness of Samples from Different Species, Regions, and Growth Years

The sweetness of RA samples from different species, regions, and growth years were obviously different (Table 1). Among the two species tested, MG produced sweeter samples than MJ. Among the five regions, samples from Shanxi (Sx) were the sweetest of all. Samples from Shaanxi (Ssx) took second place, while those from Gansu (Gs), Inner Mongolia (Nm), and Heilongjiang (Hlj) had the lowest levels of sweetness. Besides, the sweetness of RA increased as the growth period was prolonged within the range in our investigation. In addition, the increasing trend of sucrose content showed a positive correlation with the trend of RA sweetness. The increasing trend of betaine content of RA samples with five-year growth duration was also correlated with their high sweetness level. However, the betaine content of these samples started to decline after five years of growth.

### 2.2. Analysis of Content Variation Trends between the Sweetness and Four Quality Control Indicators of RA

**Figure 2:**
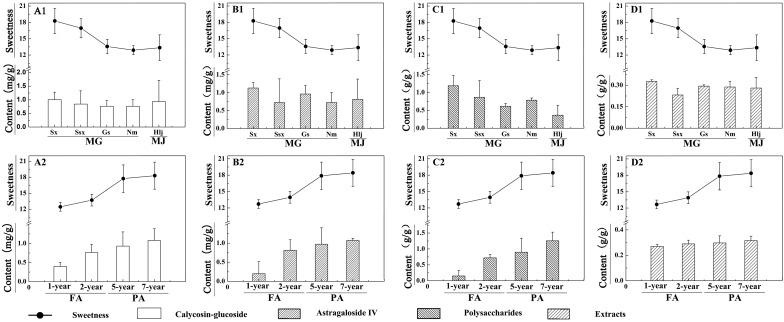
Contents of calycosin-glucoside, astragaloside IV, total polysaccharides and extracts in RA samples and RA sweetness levels across different species, regions, and growth years. Sweetness indicates relative sweetness of RA with the sweetness of 10% sucrose solution considered to be 1.0; The contents of calycosin-glucoside (mg/g), astragaloside IV (mg/g), total polysaccharides (g/g) and extracts (g/g) of RA are shown in Figure A1, A2, B1, B2, C1, C2, and D1, D2, respectively; MG (*A. membranaceus* var. Mongholicus (Bge.)) and MJ (*A. membranaceus* (Fisch.) Bge.) represent the two species of RA; FA (fast-growing RA (1–2 years samples)) and PA (perennial RA (5–7 years samples)) represent the two different growth years of RA; Sx (Shanxi province), Ssx (Shaanxi province), Gs (Gansu province), Nm (Inner Mongolia), and Hlj (Heilongjiang province) represent the five regions of RA.

Astragaloside IV, calycosin-glucoside, total polysaccharides and extracts of Chinese Pharmacopoeia (2010 Edition) are the common quality evaluation indicators of RA [14,15,16]. Therefore, we sought to determine the correlation between the sweetness level of RA and the content variation of the above four indicators. The contents of calycosin-glucoside, astragaloside IV, total polysaccharides and extracts of RA are shown in Figure 2. The variation trends of astragaloside IV, calycosin-glucoside and total polysaccharide contents of RA were similar to variation trends of their sweetness, except the content of extracts. The range of contents of extracts was very small and showed no obvious correlation with variation in RA sweetness.

### 2.3. Correlation Analysis between the Sweetness and Four Quality Control Indicators of RA

Our analysis revealed that sweetness variation in RA exhibited a strong positive correlation with calycosin-glucoside content, with a correlation coefficient of 0.659 (*p* = 0.001) (*Y*= 0.0079*X* − 0.344) (Table 2). However, sweetness variation showed only a moderate positive correlation with the contents of astragaloside IV and total polysaccharides, with correlation coefficients of 0.412 and 0.486, respectively. On the other hand, sweetness of RA exhibited only a weak negative correlation with the contents of RA extracts, with correlation coefficient of 0.183. 

Thus, sweetness of RA exhibited an obvious positive correlation with the contents of calycosin-glucoside, astragaloside IV and total polysaccharides, which is consistent with the results described above.

**Figure 3:**
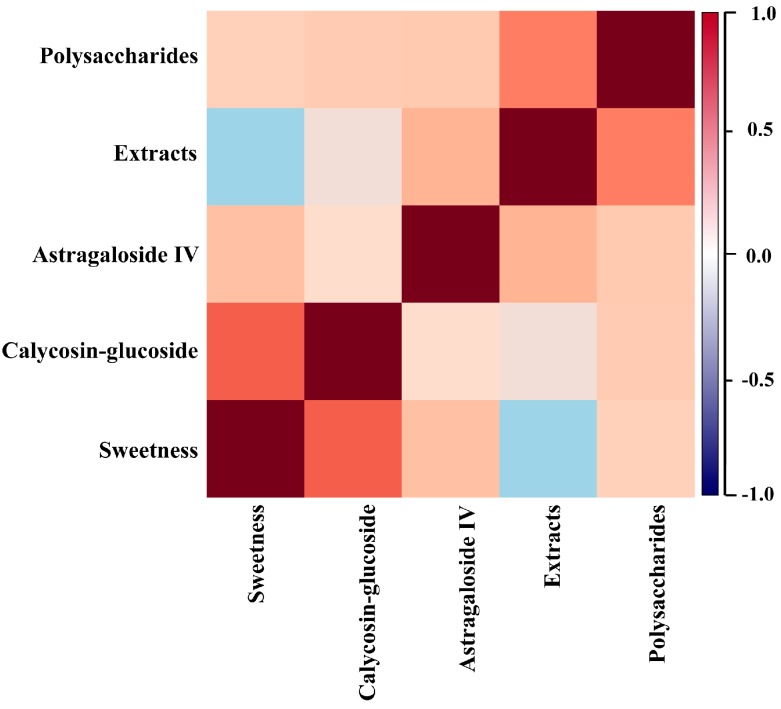
Correlation between sweetness and the contents of total polysaccharides, astragaloside IV, calycosin-glucoside, and extracts of RA. Plot showing the correlation between sweetness of RA and the contents of total polysaccharides, astragaloside IV, calycosin-glucoside, and extracts of RA; Results of correlation analysis between every pair of elements in RA are expressed in the form of bands with ranges between −1 and +1; band colors from blue (−1) to reddish brown (+1) represent correlation ranging from negative to positive. Deeper color indicates stronger correlation.

In order to further verify these results, we imported the data into Metaboanalyst 3.0 for correlation analysis of the various components of RA [17]. The results are shown in Figure 3. Sweetness levels of RA showed the highest positive correlation with calycosin-glucoside content, followed by contents of astragaloside IV and total polysaccharides. The contents of extracts and sweetness levels of RA exhibited only a weak negative correlation, which is consistent with the results of our regression analysis (Table 2).

**Table 1:** Contents of the seven components that affect RA sweetness.

	Contents	Varieties	Glc (μg/g)	Fru (μg/g)	Ino (μg/g)	Sor (μg/g)	Dul (μg/g)	Suc (mg/g)	Betaine (mg/g)	Sweetness
Batches			R.S. = 0.75	R.S. = 1.7	R.S. = 0.5	R.S. = 0.55	R.S. = 0.3	R.S. = 1.0	R.S. = 0.5	
*A. membranaceus* var. *Mongholicus* (Bge.)		Sx (*17*)	3.4 ± 1.4	1.3 ± 0.9	75.5 ± 23.6	1.5 ± 0.7	5.1 ± 1.2	17.5 ± 2.3	1.4 ± 0.7	18.3 ± 2.2
		Ssx (*7*)	1.9 ± 0.8	0.7 ± 0.5	79.0 ± 18.9	1.0 ± 0.4	4.3 ± 0.8	16.4 ± 1.8	1.4 ± 0.8	17.1 ± 1.6
		Gs (*10*)	1.0 ± 0.3	0.2 ± 0.1	79.2 ± 13.6	0.8 ± 0.2	2.9 ± 0.8	12.2 ± 1.3	2.8 ± 0.1	13.7 ± 1.3
		Nm (*8*)	0.6 ± 0.3	0.1 ± 0.1	88.4 ± 14.1	1.0 ± 0.6	2.0 ± 0.9	11.8 ± 0.8	2.2 ± 0. 9	13.7 ± 2.3
*A. membranaceus* (Fisch.) Bge.		Hlj (*6*)	0.9 ± 0.5	0.3 ± 0.2	49.0 ± 18.1	0.6 ± 0.3	2.6 ± 1.1	12.3 ± 2.4	2.2 ± 1.3	13.9 ± 0.8
Fast-growing RA		1Y (*2*)	0.7 ± 0.4	0.2 ± 0.2	38.1 ± 7.4	0.4 ± 0.2	1.9 ± 1.4	13.1 ± 0.2	0.7 ± 0.1	13.4 ± 0.5
		2Y (*18*)	0.8 ± 0.3	0.2 ± 0.1	83.3 ± 14.3	0.9 ± 0.4	2.5 ± 0.9	12.0 ± 1.1	1.4 ± 0.7	13.7 ± 1.7
Perennial RA		5Y (*22*)	2.4 ± 1.4	0.7 ± 0.4	69.3 ± 21.7	1.2 ± 0.6	4.3 ± 1.3	16.0 ± 2.8	2.5 ± 1.4	17.3 ± 2.1
		7Y (*6*)	3.8 ± 1.2	2.4 ± 0.6	91.0 ± 19.9	1.6 ± 0.7	5.4 ± 1.1	17.1 ± 0.3	2.2 ± 0.3	18.2 ± 0.5

R.S. stands for relative sweetness, which indicates the sweetness of other ingredients of the same concentration with the sweetness of 10% sucrose solution considered to be 1.0; Sx, Shanxi province; Ssx, Shaanxi province; Gs, Gansu province; Nm, Inner Mongolia; Hlj, Heilongjiang province; 1Y, 1-year-old RA; 2Y, 2-year-old RA; 5Y, 5-year-old RA; 7Y, 7-year-old RA; Glc, glucose; Fru, fructose; Ino, inositol; Sor, sorbitol; Dul, dulcitol; Suc, sucrose; Each sample was tested twice, and the average of the two values was calculated.

**Table 2:** Linear regression equations of sweetness (*X*) and the contents of polysaccharides, calycosin-glucoside, astragaloside IV, and extracts (Y) of RA.

Chemical Index	No.	Regression Equations	Related Coefficient	*p* Value
**Polysaccharides**	*48*	*Y* = 0.007*X* + 0.090	0.486	***0.147***
**Astragaloside IV**	*48*	*Y* = 0.004*X* + 0.926	0.412	***0.068***
**calycosin-glucoside**	*48*	*Y* = 0.079*X* − 0.344	0.659	***0.001***
**Extracts**	*48*	*Y* = −0.003*X* + 0.243	0.183	***0.315***

**Figure 4:**
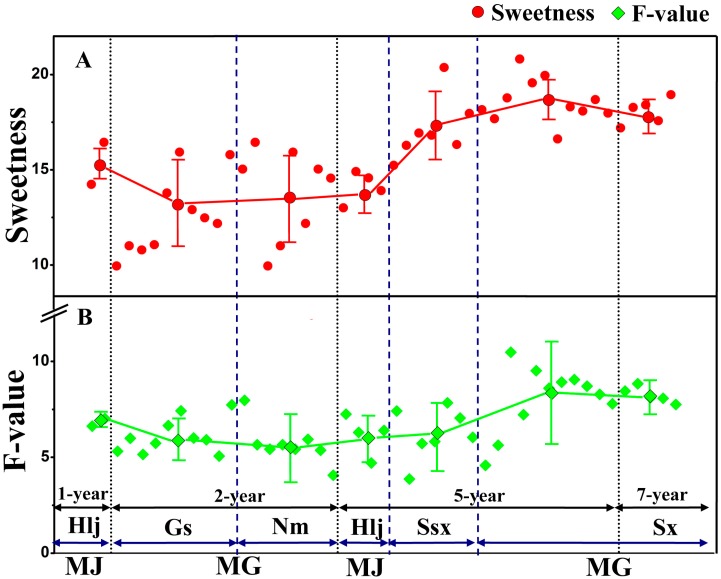
Scatter plots of sweetness (**A**) and F-value (**B**) of comprehensive evaluation of RA. Scatter plot showing the averages and degrees of dispersion of sweetness and comprehensive evaluation of RA; Sweetness indicates relative sweetness of RA with the sweetness of 10% sucrose solution considered to be 1.0; F value indicates the quality of RA with higher F value corresponding to higher integrated quality of RA; MG (*A. membranaceus* var. Mongholicus (Bge.)) and MJ (*A. membranaceus* (Fisch.) Bge.) represent the two species of RA; FA (fast-growing RA (1–2 years samples)) and PA (perennial RA (5–7 years samples)) represent the two different growth years of RA; Sx (Shanxi province), Ssx (Shaanxi province), Gs (Gansu province), Nm (Inner Mongolia), and Hlj (Heilongjiang province) represent the five regions of RA.

For a more comprehensive evaluation of RA quality, principal component analysis was applied to carry out a dimension reduction process on our dataset (Supplementary Tables S2–S4). When principal components were extracted from the components of the four indexes, we obtained principal components with an accumulated contribution rate of 89.26% (Supplementary Table S4), which could represent the information reflected in the primary data. Then the eigenvectors representing each principal component’s contribution or weight were combined, and a comprehensive evaluation function was constructed. This gave us the design formula for F, which is the value of composite evaluation index, as follows:

*F* = 43.563*X*_1_ + 27.067*X*_2_ + 6.978*X*_3_ − 2.553*X*_4_ (1)

where *X*_1_, *X*_2_, *X*_3_, *X*_4_ refer to the contents of calycosin-glucoside, astragaloside IV, total polysaccharides and extracts in RA, respectively. The coefficient in front of the variable stands for its contribution to the F value which indicates the quality of RA: the higher the F value, the better the integrated quality of RA. Data on 48 batches of RA samples were entered into the comprehensive evaluation function to obtain F values using the formula mentioned above. Details of the evaluation procedure are described in the supporting information and the results are shown in Figure 4B. The F value of Sx RA samples was obviously higher than those of other regions，and the F values of Ssx and Hlj RA samples were lower than that of Sx, while the F value of Gs RA samples was the lowest of all regions. In addition, our results indicated that F values of perennial RA (PA) samples were higher than those of fast-growing RA (FA) samples.

Figure 4 suggests that both the sweetness levels and F values increased gradually as the growing years of RA increased, which indicated the higher the RA sweetness levels, the higher the content of bioactive secondary metabolites and the better medical quality of RA.

In our research, PA samples were mostly selected from the geo-authentic producing area in Hunyuan, a country in Shanxi Province of China. The PAs used in this research were of more than 5 years of age and were planted on a slope where the soil was barren and porous. They had tolerance to sunny summers, cold winters, drought and large temperature differences between day and night. In contrast, the FA samples were transplanted in the selected managing land and harvested after two years. The PA samples suffer more environmental stress than FA samples, such as drought, cold and nutritional deficiency *etc.*

Lots of researchers have indicated that plants are resistant to stress at both the cell and whole organism level [18,19]. Plant struggle against adversity by changing in morphology, osmotic adjustment, the level of hormones, membrane protective substances, the balance of active oxygen, stress proteins and many other aspects related to water, photosynthesis, respiration, metabolism and other physiological processes in the plant [18,19,20]. It was reported that free sugars, betaine, alditols, flavonoids, saponins and other secondary metabolites were induced and accumulated at cytoplasm in respond to adversity [21,22,23]. These substances could regulate cell osmotic pressure and maintain water to enhance salt and coldness tolerance, which is vital for a plant to keep the physiological functions normal. Therefore, by suffering more environmental stresses, the level of sweetness substances and other secondary metabolites (flavonoids, saponins, polysaccharides and so on) in PA was far higher than that in FA, which might also be the reason why a strong positive correlation was observed between the traditional sweetness indicator and F values.

In addition, with the better growing environment, carbohydrates from photosynthesis in FA are prioritized to meet the demands of plant growth, such as construction of the cell skeleton, especially for the synthesis of cellulose, hemicelluloses and pectin for the plant cell walls [24,25]. By contrast, because of the environmental stress and the long growth cycles, carbohydrates from the photosynthesis of PA were mostly kept in storage and used for the synthesis of starch and other defensive secondary metabolites to resist adversity, which might explain the observation that PA had strong mealiness and sweetness characteristics instead of the stiffness and little sweetness that characterized FA.

## 3. Experimental Section

### 3.1. Plant Materials

A total of 48 RA samples were examined in this study. These samples were obtained from two species of A. *membranaceus*, and the field studies did not involve endangered or protected species. The 48 RA samples were collected from different regions, and approved by the corresponding Technology Bureaus of twenty countries in Gansu, Inner Mongolia, Shanxi, Shaanxi, and Heilongjiang provinces. Detailed information on the samples is presented in Table 3. All plant materials were identified by Xue-Mei Qin and the voucher specimens were deposited in the herbarium of the Modern Research Center for Traditional Chinese Medicine of Shanxi University. The samples were dried, ground to a fine powder, and then stored at 20 °C in a vacuum desiccator until further analysis.

### 3.2. Solvents and Chemicals

NH_4_[Cr(NCS)_4_(NH_3_)_2_]·H_2_O (ammonium Reineckate), NaHCO_3_, acetic anhydride, NaBH_4_, glacial acetic acid, phenol, methanol, ethanol, acetone, CH_2_Cl_2_, and CHCl_3_ were purchased from Fengchuan Chemical Co. Ltd. (Tianjin, China) and were of analytical grade. Acetonitrile (guaranteed grade) was bought from Thermo Fisher Lab Solutions (Shanghai, China). Ten sugar standards (d-glucose, d-galactose, d-mannose, d-fructose, d-xylose, d-arabinose, l-fucose, l-rhamnose, l-inositol, and sucrose), betaine, dimethyl sulfoxide (DMSO), and 1-methylimidazole were commercially obtained from Sigma-Aldrich China (Shanghai, China). The remaining standards of 2-deoxy-d-ribitol, rhamnitol, fucitol, arabinitol, and dulcitol were prepared by reduction of the corresponding saccharides with NaBH_4_. Astragaloside IV was commercially obtained from the National Medicine Biological Products Assay Institute (Shanghai, China) and calycosin-glucoside was bought from Shanghai Forever-Biotech Co., Ltd. (Shanghai, China)

**Table 3:** List of RA plant materials.

Species	Cultivation Pattern	Regions	No.	Harvesting Time	Growth Year
**A. *membranaceus* var. Mongholicus (Bge.)**	**FA**	Gs, Longxi County	1–3	2010.12	2
		Gs, Dangchang County	4–5	2010.12	2
		Gs, Minxian County	6–7	2011.1	2
		Gs, Weiyuan County	8–10	2011.1	2
		Nm, Guyang County	11–13	2011.1	2
		Nm, Chifeng	14–15	2011.11	2
		Nm, Ulanqab	16	2009.5	2
		Nm, Shangdu County	17	2011.11	2
		Nm, Xinghe County	18	2011	2
	**PA**	Sx, Hunyuan County	19–22	2011.11	≥5
			22–26	2011.1	5
		Sx, Daixian County	27–28	2011.1	5
		Sx, Yinxian County	29–31	2011.9	5
			32	2011.1	≥5
		Sx, Tianzhen County	33	2011.1	5
		Sx, Yanggao County	34	2011.1	5
		Sx, Xinzhou	35	2011.9	5
		Ssx, Zizhou County	36	2011.9	6
		Ssx	37	2009.6	5
		Ssx, Yulin	38–42	2011	5
**A. *membranaceus* (Fisch.) Bge*.***	**FA**	Hlj, Hulan County	43	2011.1	1
		Hlj, Hulan County	44	2011	1
	**PA**	Hlj, Hulan County	45–47	2011.11	5
		Hlj, Jiagedaqi	48	2011	5

### 3.3. Apparatus

The apparatus used were as follows: gas chromatography-mass spectrometer (Thermo Finnigan Ion trap GC/MS, Shanghai, China), Dionex UltiMate 3000 HPLC (Shanghai, China), Alltech 2000 ES evaporative light scattering detector (ELSD) ( Shanghai, China), ultraviolet-visible spectrophotometer (UV-7501, Wuxi Kedeng Instrument Plant, Wuxi, China), vacuum freeze-drier (DZF-1B, Shanghai Yuejin Medical Instrument Plant, Shanghai, China), rotary evaporator RE-52A (Shanghai Yarong Biochemical Instrument Plant, Shanghai, China), high-speed refrigerated centrifuge (TGL-16 type, Ningbo Scientz Biotechnology Co., Ltd., Ningbo, China), and desk-top constant temperature shaking table (SHK-99-II type, Beijing North TZ-Biotech Develop, Co., Ltd. Beijing, China).

### 3.4. Determination of Monosaccharide, Disaccharide, and Alditol Contents in RA by GC-MS

Derivatization of RA samples was done following the previously described method [26]. This method was concurrently used to generate the GC-MS chromatogram of the 22 monosaccharide, disaccharide, and alditol standard substances. Briefly, fine powdered RA (50 mg) was placed into a 10 mL centrifuge tube. Then, chloroform-methanol-water solution (1 mL, 12:5:1 by volume) was added in order to extract the powder. After allowing the sample to fully soak the solution with 100 μg/mL ribitol was shaken and extracted at 4 °C. The solution was centrifuged, and the supernatant was evaporated. The sample was redissolved into 100 μL of DMSO, and 1-methylimidazole was added as catalyst. Acetic anhydride was added as the acetylation reagent. After letting the derivatization reaction run for 10 min, the solution was added to 1 mL of ddH_2_O to terminate the reaction and 500 μL of CH_2_Cl_2_ was used for extracting twice. The solution was then combined with the organic phase. After drying, the sample was redissolved into 200 μL of CH_2_Cl_2_ for filtering and a 1 μL sample was obtained and subjected to GC/MS.

### 3.5. Determination of Betaine and Total Polysaccharide Contents in RA by UV-Vis

#### 3.5.1. Betaine

The standard curve of betaine content in RA was obtained according to a previously described method [27]. Approximately 1 g of dried RA powder was added to 25 mL of 80% methanol. After reflux extraction at 70–75 °C for 1 h, the solution was cooled and filtered. Approximately 10 mL of 80% methanol was divided into three parts for washing the residue. The solution was then filtered. The filtrate was combined with the washes and then concentrated to 5 mL. After adjusting the pH of the solution to 1.0 with 35% HCl, 0.5 g of active carbon was added and heated until boiling, then cooled and filtered. The sample was washed with 10 mL of ddH_2_O divided into three parts, and the washes and filtrates were combined. Approximately 10 mL of fresh 2.5% Reinecke salt were added. The solution was cooled at 5 °C for 30 min, centrifuged at 4000 r/min for 15 min, and then precipitated. After the supernatant was discarded, 15 mL of 99% diethyl ether was added and centrifuged at 4000 r/min for 15 min. The supernatant was concentrated until the diethyl ether was completely evaporated, and 70% acetone was used for dissolution and precipitation. The solution was transferred into a volumetric flask, which was used to adjust the volume to 50 mL. A blank control was prepared by a similar method using ddH_2_O. Absorbance was determined at 525 nm, and the betaine content in RA was calculated based on the standard curve.

#### 3.5.2. Polysaccharides

Anhydrous glucose was used as control to determine the polysaccharide content of RA using the phenol-sulfuric acid method [28]. The processing method for the plant sample was as follows: 1 g of fine RA powder was placed in a 250 mL round-bottom flask and mixed with 100 mL of water. The solution was extracted under reflux for 1 h, and the supernatant was recovered. The extraction procedure was performed twice, and the two filtrates were combined and then centrifuged. After concentration, the solution was transferred into a 1 KD dialysis membrane and dialyzed thrice. The dialyzate was freeze-dried every 12 h, and the total solid polysaccharide content was obtained. The total polysaccharides were weighed and used to prepare polysaccharide solutions ranging in concentration from 50 ng/mL to 150 ng/mL. Approximately 2 mL of the solution was placed into a test tube and subjected to the phenol-sulfuric acid method. Absorbance was determined at 488 nm, and the total polysaccharide content of the RA samples was determined according to the standard curve. ddH_2_O (2 mL) was used as blank.

### 3.6. Determination of Astragaloside IV and Calycosin-Glucoside in RA by HPLC

#### 3.6.1. Astragaloside IV

The content of astragaloside IV was determined as described previously [29]. Astragaloside IV was weighed and used to prepare a series of standard solutions of different concentrations. HPLC-ELSD was used to obtain the chromatogram of the control substances. A standard curve equation was obtained by calculating the log of sample amount (μg) or the log of peak area.

Approximately 4 g of the fine RA powder was placed in a 250 mL conical flask to which 150 mL of methanol and 15 mL of ammonium hydroxide were successively added. The solution was preserved hermetically closed after 60 min of ultrasound treatment. Extracts were filtered and washed three times with 90 mL of methanol. The pooled supernatant was evaporated to dryness under vacuum. Then, the dried powder extracts were dissolved in 10 mL of water to pass through D101 macro-porous adsorptive resin (inner 1.5 cm × 12 cm). Elution was performed using 50 mL of water, 80 mL of 30% ethanol, and 120 mL of 95% ethanol in sequence. The 95% ethanol eluant was collected and dried. After dissolving in methanol, a volumetric flask was used to adjust the volume to 5 mL. The subsequent filtrate of the supernatant was collected for detection, and the data was entered into the standard curve equation to calculate the content of astragaloside IV in the RA samples.

#### 3.6.2. Calycosin-Glucoside

The content of calycosin-glucoside was determined according to a previously described method [29]. Calycosin-glucoside was weighed and used to prepare standard solutions of different concentrations. HPLC-UV-Vis was used to detect and obtain the chromatogram of the control substances. A standard curve equation was obtained by calculating the log of sample amount (μg) or the log of peak area.

Approximately 1 g of the fine RA powder was weighed, placed in a 100 mL round-bottom flask, and mixed with 50 mL of methanol. The solution was weighed, heated in a water bath, and reflux extracted for 4 h. The solution was cooled to room temperature and then weighed again. Any weight loss was compensated by adding methanol, and the solution was mixed and filtered. Approximately 25 mL of the subsequent filtrate was carefully obtained. The solvent removed until the sample was dry. After dissolving in methanol, a volumetric flask was used to adjust the final volume to 5 mL. The subsequent filtrate was collected for detection, and the data was entered into the standard curve equation to determine the content of calycosin-glucoside in the RA samples.

### 3.7. Determination of the Content of Aqueous Extracts in RA

A total of 4 g of fine RA powder was accurately weighed and placed in a 250 mL conical flask, into which 100 mL of ddH_2_O was poured. After sealing, cold quenching and shaking for 6 consecutive hours, and stewing for 18 h, the solution was filtered rapidly with a dry filter. Then, 20 mL of the resulting filtrate was put it into an evaporating dish and dried to constant weight. The weight of the dried residue was used to calculate the content (%) of the extracts in RA.

### 3.8. Calculation of Sweetness

“Sweetness” was used as an index to characterize the strength of sweetness components in RA. Specific sweetness of various components of sweetener values, including Calories and Glycemic Index were obtained from the website sugar and sweetener guide [30]. Total sweetness was calculated using the following formula:

$$S_{\left(i\right)}=\sum_{i=1}^{n}T_{i}\times c_{i}$$

where n is the total number of sweet components in RA, *T_i_* indicates the relative sweetness of the sweet components when the sweetness of sucrose is defined as 1.0, and *c_i_* represents the content of other sweet components in RA.

The evaluation index could be used to characterize the total sweetness of all components of monosaccharides, disaccharides, alditols, and betaine in RA. These were the main constituents that contributed to the sweetness of RA.

### 3.9. Statistical Analysis

Correlation analyses for all data (Mean ± SD) were performed using SPSS 16.0. Correlation analysis of sweetness and the four quality control indicator were conducted through the website of metaboanalyst [17]. Origin 8.0 was used to make all figures.

## 4. Conclusions

RA has traditionally been used for tonifying the spleen and blood and for vital energy [31,32,33]. Today it is used as an antiperspirant, a diuretic, or a tonic in Oriental medicines in many Asian countries. Moreover, it is popularly used in functional foods and nutraceuticals in Western countries [34]. Such extensive application of RA depends highly on its quality, and in consequence screening better evaluation indicators is an important task for its safe usage [35,36,37,38]. Our results indicate that sweetness is one of the most important quality attributes of RA and that the traditional sensory indicator of sweetness can be used as a reliable reference for RA quality. Thus, our findings provide important insights into the usefulness of sweetness as a quality indicator of this Chinese herb.

## Supplementary Materials

Supplementary materials can be accessed at: http://www.mdpi.com/1420-3049/20/02/3129/s1.

## Abbreviation

RA: Radix Astragali
MG: *A. membranaceus* var. Mongholicus (Bge.), and MJ, *A. membranaceus* (Fisch.) Bge., represent the two species of RA
FA: fast-growing RA, and PA, perennial RA, represent the two different growth years of RA
Sx: Shanxi Province
Ssx: Shaanxi Province
Gs: Gansu Province
Nm: Inner Mongolia
Hlj: Heilongjiang Province
GC-MS: gas chromatography-mass spectrometry
UV-Vis: ultraviolet visible
DMSO: dimethyl sulfoxide
HPLC: high-performance liquid chromatography
ELSD: evaporative light scattering detector
