# Supplementary material for: Potential Quality Evaluation Method for Radix Astragali Based on Sweetness Indicators
Source: Molecules. 2015 Feb 13;20(2):3129–45. doi: 10.3390/molecules20023129 (PMC6272557; doi:10.3390/molecules20023129)
Supplement: Supplementary file 1 [file molecules-20-03129-s001.pdf]

Supplementary Materials

Calculation of RA sweetness:

Step 1: The main components contributing to sweetness of RA were determined. The results are shown in Table S1.

Table S1. Qualitative and quantitative analysis of sweetness of RA.

No.	Glc (μg/g) R.S. = 0.75	Fru (μg/g) R.S. = 1.7	Ino (μg/g) R.S. = 0.5	Sor (μg/g) R.S. = 0.55	Dul (μg/g) R.S. = 0.3	Suc (mg/g) R.S. = 1.0	Betaine (mg/g) R.S. = 0.5
**1**	0.773 ± 0.122	2.98 ± 0.027	12.348 ± 3.983	6.116 ± 1.658	7.46 ± 0.245	11.204 ± 1.991	2.55 ± 0.333
**2**	1.955 ± 0.104	ND	9.408 ± 5.759	0.636 ± 0.74	3.602 ± 0.12	14.02 ± 1.292	2.03 ± 0.663
**3**	1.659 ± 0.116	0.98 ± 0.028	46.573 ± 0.998	3.344 ± 0.24	6.937 ± 0.077	11.012 ± 1.216	3.793 ± 0.183
**4**	0.994 ± 0.342	0.102 ± 0.013	10.096 ± 4.724	2.414 ± 1.514	3.512 ± 0.247	14.001 ± 1.179	3.587 ± 0.628
**5**	0.248 ± 0.016	0.102 ± 0.039	3.363 ± 5.144	2.346 ± 1.433	4.092 ± 0.553	14.173 ± 0.582	2.32 ± 0.447
**6**	0.181 ± 0.335	0.143 ± 0.044	4.117 ± 4.45	0.25 ± 0.26	2.456 ± 0.051	12.174 ± 0.582	2.556 ± 0.447
**7**	0.804 ± 0.122	0.454 ± 0.028	1.529 ± 0.793	1.309 ± 0.085	1.8 ± 0.022	11.066 ± 1.143	3.681 ± 0.887
**8**	1.218 ± 0.116	0.758 ± 0.038	21.162 ± 5.281	0.853 ± 0.789	1.837 ± 0.21	12.071 ± 0.997	1.325 ± 0.036
**9**	0.803 ± 0.335	0.312 ± 0.011	40.371 ± 5.894	8.564 ± 1.309	10.275 ± 0.804	11.105 ± 1.329	2.15 ± 0.552
**10**	1.326 ± 0.122	0.146 ± 0.028	14.793 ± 6.628	1.792 ± 0.25	4.265 ± 0.181	13.512 ± 0.699	2.105 ± 0.567
**11**	0.553 ± 0.317	0.469 ± 0.02	26.217 ± 3.306	4.491 ± 3.344	2.261 ± 1.346	13.099 ± 1.487	1.621 ± 1.142
**12**	0.656 ± 0.204	0.091 ± 0.032	19.734 ± 2.269	3.281 ± 5.354	3.326 ± 1.514	14.158 ± 2.013	0.826 ± 3.271
**13**	0.901 ± 0.104	0.057 ± 0.002	27.976 ± 3.191	0.74 ± 1.219	4.731 ± 2.101	12.438 ± 0.787	2.611 ± 0.333
**14**	0.865 ± 0.301	0.026 ± 0.016	20.616 ± 3.433	5.039 ± 3.444	3.305 ± 0.789	14.426 ± 1.195	0.958 ± 1.017
**15**	1.509 ± 0.282	0.752 ± 0.05	28.492 ± 2.237	7.421 ± 2.288	10.553 ± 1.325	12.113 ± 1.137	1.783 ± 0.003
**16**	1.521 ± 0.162	0.768 ± 0.011	6.943 ± 1.778	1.128 ± 0.467	5.556 ± 0.097	10.03 ± 1.44	2.07 ± 0.001
**17**	1.05 ± 0.158	0.314 ± 0.006	16.253 ± 5.264	4.418 ± 0.26	19.868 ± 2.661	12.328 ± 0.877	3.816 ± 0.183
**18**	0.116 ± 0.077	0.023 ± 0.014	10.994 ± 1.532	4.418 ± 0.976	0.538 ± 0.093	9.953 ± 0.546	2.035 ± 0.149
**19**	1.473 ± 0.122	0.058 ± 0.016	25.802 ± 7.318	3.29 ± 1.8	2.542 ± 0.252	13.733 ± 0.997	4.385 ± 0.257
**20**	0.116 ± 0.097	0.056 ± 0.048	1.16 ± 3.199	1.433 ± 0.467	0.447 ± 0.248	12.178 ± 1.366	3.211 ± 0.22
**21**	2.101 ± 0.238	0.338 ± 0.017	39.393 ± 1.176	1.219 ± 0.24	6.982 ± 1.05	10.027 ± 0.847	1.974 ± 0.22
**22**	0.144 ± 0.051	0.803 ± 0.015	2.869 ± 0.842	5.199 ± 3.326	1.035 ± 0.689	14.155 ± 0.847	2.574 ± 0.11
**23**	2.792 ± 0.22	ND	37.028 ± 8.412	2.954 ± 0.465	6.599 ± 0.558	9.43 ± 1.29	2.724 ± 0.776
**24**	0.523 ± 0.236	0.226 ± 0.015	4.031 ± 1.544	3.29 ± 1.128	12.071 ± 1.521	12.013 ± 0.699	2.037 ± 0.567
**25**	0.6 ± 0.225	0.203 ± 0.049	6.786 ± 5.031	2.264 ± 4.93	4.785 ± 4.705	17.654 ± 0.732	0.621 ± 2.605
**26**	0.245 ± 0.171	0.076 ± 0.05	3.59 ± 4.174	1.658 ± 3.937	4.564 ± 4.69	16.157 ± 0.381	2.342 ± 0.957
**27**	2.177 ± 0.059	1.161 ± 0.014	46.315 ± 4.524	3.933 ± 9.077	34.564 ± 3.767	17.589 ± 0.44	1.567 ± 0.123
**28**	0.44 ± 0.116	0.102 ± 0.011	10.813 ± 6.618	1.271 ± 2.004	11.16 ± 7.236	15.761 ± 0.319	2.115 ± 1.436

Table S1. *Cont.*

No.	Glc (μg/g) R.S. = 0.75	Fru (μg/g) R.S. = 1.7	Ino (μg/g) R.S. = 0.5	Sor (μg/g) R.S. = 0.55	Dul (μg/g) R.S. = 0.3	Suc (mg/g) R.S. = 1.0	Betaine (mg/g) R.S. = 0.5
**29**	1.689 ± 0.263	0.242 ± 0.017	12.596 ± 4.884	3.326 ± 0.338	6.54 ± 1.933	16.354 ± 0.177	1.163 ± 1.406
**30**	0.558 ± 0.274	0.248 ± 0.011	10.331 ± 4.1	2.177 ± 3.933	8.541 ± 0.465	15.104 ± 0.991	2.354 ± 0.782
**31**	0.531 ± 0.089	0.152 ± 0.043	7.864 ± 3.937	6.543 ± 2.668	16.553 ± 0.976	15.096 ± 1.108	4.276 ± 0.477
**32**	2.589 ± 0.176	0.129 ± 0.023	67.166 ± 4.44	3.71 ± 1.856	7.931 ± 1.595	18.529 ± 0.44	0.831 ± 0.087
**33**	1.325 ± 0.031	0.698 ± 0.041	35.997 ± 5.407	26.217 ± 2.414	18.562 ± 0.994	16.619 ± 0.732	2.29 ± 0.093
**34**	4.69 ± 0.216	ND	32.051 ± 4.796	3.937 ± 6.116	28.616 ± 0.773	17.83 ± 1.491	0.946 ± 0.027
**35**	2.661 ± 0.065	0.357 ± 0.039	66.47 ± 5.989	5.199 ± 2.264	16.924 ± 0.6	16.235 ± 0.761	0.913 ± 0.423
**36**	4.705 ± 0.059	0.203 ± 0.044	45.472 ± 5.213	4.93 ± 1.8	67.719 ± 1.473	18.681 ± 0.79	2.553 ± 0.512
**37**	5.039 ± 0.278	1.758 ± 0.014	87.085 ± 4.088	21.162 ± 1.792	36.454 ± 1.326	18.727 ± 0.761	1.688 ± 1.412
**38**	7.236 ± 0.086	1.855 ± 0.036	86.481 ± 5.309	8.004 ± 2.954	52.532 ± 2.792	19.189 ± 0.148	1.26 ± 0.063
**39**	3.281 ± 0.462	1.668 ± 0.044	46.249 ± 3.8	5.354 ± 3.71	35.124 ± 2.589	18.075 ± 0.644	0.667 ± 0.363
**40**	4.491 ± 0.912	0.679 ± 0.032	111.769 ± 5.358	20.616 ± 3.344	18.102 ± 1.659	16.241 ± 0.936	2.066 ± 0.423
**41**	6.543 ± 0.316	3.009 ± 0.028	70.262 ± 4.619	19.734 ± 1.559	35.369 ± 2.103	17.79 ± 2.192	1.976 ± 0.093
**42**	0.517 ± 0.082	ND	22.774 ± 4.917	4.256 ± 1.421	14.91 ± 1.509	16.151 ± 0.732	2.099 ± 0.602
**43**	3.767 ± 0.766	2.514 ± 0.045	90.073 ± 4.299	9.077 ± 2.955	58.83 ± 3.708	16.777 ± 0.381	2.401 ± 0.213
**44**	1.933 ± 0.038	1.323 ± 0.032	33.567 ± 4.331	27.976 ± 0.636	42.429 ± 1.955	17.837 ± 0.703	1.694 ± 0.363
**45**	3.708 ± 0.036	1.131 ± 0.036	74.842 ± 5.629	2.955 ± 1.271	5.346 ± 0.44	16.201 ± 0.261	2.963 ± 0.777
**46**	2.103 ± 0.125	0.6 ± 0.04	37.598 ± 7.342	11.559 ± 4.256	36.658 ± 0.517	17.76 ± 1.17	0.659 ± 0.063
**47**	1.595 ± 0.569	0.308 ± 0.029	15.739 ± 5.054	1.856 ± 8.564	8.999 ± 0.803	16.446 ± 1.75	2.311 ± 0.542
**48**	1.925 ± 0.034	0.159 ± 0.013	7.596 ± 0.134	1.68 ± 0.096	5.264 ± 0.234	13.57 ± 0.017	4.179 ± 0.099

ND means 'not detected'.

Step 2: The equation used to calculate the integrated sweetness of the seven components is indicated below:

$$S_{(i)} = SUM\ (T_i * c_i)$$

where T_i indicates relative sweetness of the components with the sweetness of 10% sucrose defined as 1.0, and c_i represents the content of other sweet components in RA.

Thus, the formula of integrated RA sweetness was obtained as follows:

$$S_{(i)} = 0.75{*}c_{Glc} + 1.7{*}c_{Fru} + 0.5c_{Ino} + 0.55{*}c_{Sor} + 0.3{*}c_{Dul} + 1{*}c_{Suc} + 0.5{*}c_{betaine}$$

Analytical procedure for comprehensive quality evaluation of RA samples:

Step 1: The original experimental data, including the contents of calycosin-glycoside, astragaloside IV, polysaccharides, and extracts in RA samples, were calculated.

Table S2. Raw data on the contents of calycosin-glycoside, astragaloside IV, polysaccharides, and extracts in RA samples（Mean ± SD).

No.	Calycosin-glycoside (mg/g)	Astragaloside IV (mg/g)	Polysaccharides (g/g)	Extracts (g/g)
**1**	0.76 ± 0.006	0.23 ± 0.034	0.043 ± 0.001	0.24 ± 0.012
**2**	0.89 ± 0.023	0.77 ± 0.01	0.102 ± 0.01	0.425 ± 0.035
**3**	1.2 ± 0.009	1.23 ± 0.023	0.171 ± 0.002	0.357 ± 0.017
**4**	0.67 ± 0.017	0.34 ± 0.025	0.131 ± 0.02	0.321 ± 0.021
**5**	0.53 ± 0.02	0.68 ± 0.039	0.104 ± 0.006	0.357 ± 0.009
**6**	0.32 ± 0.011	0.89 ± 0.014	0.047 ± 0	0.314 ± 0.011
**7**	0.53 ± 0.009	0.7 ± 0.024	0.114 ± 0.004	0.389 ± 0.019
**8**	0.37 ± 0.034	0.69 ± 0.003	0.056 ± 0.002	0.363 ± 0.006
**9**	1.55 ± 0.187	0.93 ± 0.024	0.095 ± 0.004	0.338 ± 0.013
**10**	0.7 ± 0.13	1.15 ± 0.043	0.072 ± 0.005	0.285 ± 0.011
**11**	0.56 ± 0.011	0.7 ± 0.027	0.072 ± 0.004	0.303 ± 0.001
**12**	0.58 ± 0.008	1.04 ± 0.034	0.111 ± 0.001	0.293 ± 0.009
**13**	0.73 ± 0.265	0.85 ± 0.02	0.084 ± 0.007	0.253 ± 0.025
**14**	0.81 ± 0.057	0.63 ± 0.013	0.133 ± 0.012	0.286 ± 0.016
**15**	1.03 ± 0.112	1.18 ± 0.028	0.101 ± 0.003	0.316 ± 0.021
**16**	1.3 ± 0.131	0.69 ± 0.079	0.122 ± 0.007	0.335 ± 0.02
**17**	0.51 ± 0.1	1.5 ± 0.019	0.102 ± 0.004	0.329 ± 0.006
**18**	0.63 ± 0.137	0.61 ± 0.014	0.073 ± 0.005	0.374 ± 0.008
**19**	0.31 ± 0.253	1.16 ± 0.002	0.094 ± 0.001	0.32 ± 0.006
**20**	0.77 ± 0.034	0.89 ± 0.042	0.102 ± 0.008	0.322 ± 0.011
**21**	0.63 ± 0.022	1.3 ± 0.002	0.104 ± 0.001	0.231 ± 0.003
**22**	0.59 ± 0.035	0.88 ± 0.097	0.102 ± 0.005	0.378 ± 0.004
**23**	0.46 ± 0.071	1.42 ± 0.013	0.144 ± 0.013	0.216 ± 0.008
**24**	0.28 ± 0.092	2.13 ± 0.011	0.146 ± 0.006	0.297 ± 0.014
**25**	1.22 ± 0.074	0.17 ± 0.058	0.134 ± 0.009	0.263 ± 0.018
**26**	1.1 ± 0.008	0.72 ± 0.185	0.142 ± 0.018	0.267 ± 0.008
**27**	1.12 ± 0.033	1.08 ± 0.055	0.075 ± 0.011	0.191 ± 0.012
**28**	0.86 ± 0.003	0.56 ± 0.023	0.143 ± 0.019	0.171 ± 0.02
**29**	1.03 ± 0.221	0.57 ± 0.031	0.053 ± 0.001	0.267 ± 0.015
**30**	0.37 ± 0.002	0.69 ± 0.034	0.056 ± 0.002	0.333 ± 0.022
**31**	1.55 ± 0.026	0.97 ± 0.047	0.095 ± 0.003	0.338 ± 0.016
**32**	1.3 ± 0.008	1.2 ± 0.063	0.189 ± 0.007	0.328 ± 0.011
**33**	1.1 ± 0.194	1.11 ± 0.051	0.116 ± 0.006	0.32 ± 0.023
**34**	1.21 ± 0.031	1.16 ± 0.048	0.102 ± 0.005	0.331 ± 0.037

Table S2. *Cont.*

No.	Calycosin-glycoside (mg/g)	Astragaloside IV (mg/g)	Polysaccharides (g/g)	Extracts (g/g)
**35**	1.37 ± 0.004	1.06 ± 0.042	0.1 ± 0.001	0.323 ± 0.005
**36**	1.48 ± 0.065	1.03 ± 0.021	0.101 ± 0.005	0.348 ± 0.013
**37**	1.23 ± 0.01	1.18 ± 0.064	0.172 ± 0.002	0.327 ± 0.028
**38**	1.24 ± 0.167	1.21 ± 0.053	0.107 ± 0.01	0.321 ± 0.01
**39**	1.23 ± 0.177	1.51 ± 0.066	0.128 ± 0.023	0.322 ± 0.016
**40**	1.02 ± 0.053	1.06 ± 0.004	0.101 ± 0.019	0.313 ± 0.039
**41**	1.72 ± 0.011	1.07 ± 0.026	0.134 ± 0.006	0.332 ± 0.025
**42**	0.88 ± 0.023	0.78 ± 0.003	0.074 ± 0.02	0.327 ± 0.123
**43**	0.37 ± 0.079	1.22 ± 0.042	0.076 ± 0.03	0.339 ± 0.041
**44**	1.12 ± 0.04	1.01 ± 0.028	0.131 ± 0.003	0.302 ± 0.011
**45**	1.22 ± 0.003	1.07 ± 0.047	0.106 ± 0.002	0.343 ± 0.02
**46**	1.19 ± 0.086	1.16 ± 0.053	0.089 ± 0.001	0.328 ± 0.022
**47**	1.29 ± 0.084	1.18 ± 0.008	0.124 ± 0.005	0.331 ± 0.003
**48**	1.22 ± 0.09	1.24 ± 0.03	0.092 ± 0.007	0.337 ± 0.011

Step 2: The original experimental data were standardized by SPSS 16.0 for principal component analysis, and the results are shown in Table S3.

Table S3. Standardized information of the data in Table S2.

No.	Calycosin-glycoside	Astragaloside IV	Polysaccharides	Extracts	No.	Calycosin-glycoside	Astragaloside IV	Polysaccharides	Extracts
**1**	−0.419	−2.129	−1.935	−1.518	**20**	−0.393	−0.232	−0.109	0.177
**2**	−0.078	−0.577	−0.109	2.307	**21**	−0.761	0.946	−0.047	−1.704
**3**	0.735	0.745	2.027	0.901	**22**	−0.866	−0.261	−0.109	1.335
**4**	−0.656	−1.813	0.789	0.157	**23**	−1.207	1.291	1.191	−2.014
**5**	−1.023	−0.836	−0.047	0.901	**24**	−1.679	3.331	1.253	−0.339
**6**	−1.574	−0.232	−1.811	0.012	**25**	0.788	−2.301	0.881	−1.042
**7**	−1.023	−0.778	0.262	1.563	**26**	0.473	−0.721	1.129	−0.960
**8**	−1.443	−0.807	−1.533	1.025	**27**	0.525	0.314	−0.945	−2.531
**9**	1.654	−0.117	−0.326	0.508	**28**	−0.157	−1.181	1.160	−2.944
**10**	−0.577	0.515	−1.037	−0.587	**29**	0.289	−1.152	−1.625	−0.960
**11**	−0.944	−0.778	−1.037	−0.215	**30**	−1.443	−0.807	−1.533	0.405
**12**	−0.892	0.199	0.170	−0.422	**31**	1.654	−0.002	−0.326	0.508
**13**	−0.498	−0.347	−0.666	−1.249	**32**	0.998	0.658	2.584	0.301
**14**	−0.288	−0.979	0.850	−0.567	**33**	0.473	0.400	0.324	0.136
**15**	0.289	0.601	−0.140	0.053	**34**	0.762	0.544	−0.109	0.363
**16**	0.998	−0.807	0.510	0.446	**35**	1.182	0.256	−0.171	0.198
**17**	−1.076	1.521	−0.109	0.322	**36**	1.470	0.170	−0.140	0.715
**18**	−0.761	−1.037	−1.006	1.252	**37**	0.814	0.601	2.057	0.281
**19**	−1.600	0.544	−0.357	0.136	**38**	0.840	0.687	0.046	0.157

Table S3. *Cont.*

No.	Calycosin-glycoside	Astragaloside IV	Polysaccharides	Extracts	No.	Calycosin-glycoside	Astragaloside IV	Polysaccharides	Extracts
**39**	0.814	1.549	0.696	0.177	**44**	0.525	0.113	0.789	−0.236
**40**	0.263	0.256	−0.140	−0.009	**45**	0.788	0.285	0.015	0.612
**41**	2.100	0.285	0.881	0.384	**46**	0.709	0.544	−0.511	0.301
**42**	−0.104	−0.548	−0.976	0.281	**47**	0.972	0.601	0.572	0.363
**43**	−1.443	0.716	−0.914	0.529	**48**	0.788	0.773	−0.418	0.488

Step 3: Principal component analysis was applied to carry out a dimension reduction process of the standardizing date and the results are shown in Table S4.

Table S4. Eigenvalue, contribution rate, accumulated contribution rate and eigenvector of principle component analysis.

Component	Initial Eigenvalues			Eigenvector			
	Total	% of Variance	Cumulative %	X_1	X_2	X_3	X_4
C_1	1.327	33.165	33.165	0.4566	0.4132	0.5174	0.5938
C_2	1.207	30.187	63.352	0.3486	0.6881	−0.5953	−0.2276
C_3	1.036	25.911	89.263	0.6907	−0.2859	0.3006	−0.5934

C_1, C_2, C_3 indicate the principal components 1, 2, and 3, respectively; X_1, X_2, X_3, X_4 indicate calycosin-glycoside, astragaloside IV, polysaccharides, and extracts, respectively.

The linear equations of the three principal components based on the eigenvectors are indicated below:

$$C_1 = 0.4566\,X_1 + 0.4132\,X_2 + 0.5174\,X_3 + 0.5938\,X_4$$
$$C_2 = 0.3486\,X_1 + 0.6881\,X_2 - 0.5953\,X_3 - 0.2276\,X_4$$
$$C_3 = 0.6907\,X_1 - 0.2859\,X_2 + 0.3006\,X_3 - 0.5934\,X_4$$

Step 4: The contribution of each principal component was used to derive the comprehensive evaluation function as described below:

$$\begin{aligned} F &= 33.165\,C_1 + 30.187\,C_2 + 25.911\,C_3 \\ &= 33.165 * (0.4566\,X_1 + 0.4132\,X_2 + 0.5174\,X_3 + 0.5938\,X_4) \\ &+ 30.187 * (0.3486\,X_1 + 0.6881\,X_2 - 0.5953\,X_3 - 0.2276\,X_4) \\ &+ 25.911 * (0.6907\,X_1 - 0.2859\,X_2 + 0.3006\,X_3 - 0.5934\,X_4) \end{aligned}$$

The above analysis shows that the design formula for F can be expressed as follows:

$$F = 43.563\,X_1 + 27.067\,X_2 + 6.978\,X_3 - 2.553\,X_4$$
